# Evidence for diagnosis of early chronic pancreatitis after three episodes of acute pancreatitis: a cross-sectional multicentre international study with experimental animal model

**DOI:** 10.1038/s41598-020-80532-6

**Published:** 2021-01-14

**Authors:** Péter J. Hegyi, Alexandra Soós, Emese Tóth, Attila Ébert, Viktória Venglovecz, Katalin Márta, Péter Mátrai, Alexandra Mikó, Judit Bajor, Patrícia Sarlós, Áron Vincze, Adrienn Halász, Ferenc Izbéki, Zoltán Szepes, László Czakó, György Kovács, Mária Papp, Zsolt Dubravcsik, Márta Varga, József Hamvas, Balázs C. Németh, Melania Macarie, Ali Tüzün Ince, Dmitry S. Bordin, Elena A. Dubtsova, Mariya A. Kiryukova, Igor E. Khatkov, Tanya Bideeva, Artautas Mickevicius, Elena Ramírez-Maldonado, Ville Sallinen, Bálint Erőss, Dániel Pécsi, Andrea Szentesi, Andrea Párniczky, László Tiszlavicz, Péter Hegyi

**Affiliations:** 1 Institute for Translational Medicine, Medical School, Szentágothai Research Centre, University of Pécs, Pécs, Hungary; 2 Division of Gastroenterology, First Department of Medicine, Medical School, University of Pécs, Pécs, Hungary; 3 Department of Gastroenterology, Slovak Medical University in Bratislava, Bratislava, Slovakia; 4 Department of Medicine, University of Szeged, Szeged, Hungary; 5 Department of Pharmacology and Pharmacotherapy, Szeged, Hungary; 6 Szent György Teaching Hospital of County Fejér, Székesfehérvár, Hungary; 7 Department of Internal Medicine, Division of Gastroenterology, University of Debrecen, Debrecen, Hungary; 8 Bács-Kiskun County Hospital, Kecskemét, Hungary; 9 Dr. Réthy Pál Hospital, Békéscsaba, Hungary; 10 Peterfy Hospital and Trauma, Trauma Emergency Room, Esztergom, Hungary; 11 County Emergency Clinical Hospital - Gastroenterology and, University of Medicine, Pharmacy, Sciences and Technology, Târgu Mureș, Romania; 12 School of Medicine, Hospital of Bezmialem Vakif University, Istanbul, Turkey; 13 A.S. Loginov Moscow Clinical Scientific Center, Moscow, Russia; 14 Tver State Medical University, Tver, Russia; 15 A.I. Yevdokimov Moscow State University of Medicine and Dentistry, Moscow, Russia; 16 Semashko Central Clinical Hospital, Moscow, Russia; 17 Clinic of Gastroenterology, Nephrourology and Abdominal Surgery, Faculty of Medicine, Vilnius University, Vilnius,, Lithuania; 18 Consorci Sanitari del Garraf, Sant Pere de Ribes, Barcelona, Spain; 19 Department of Abdominal Surgery, University of Helsinki and Helsinki University Hospital, Helsinki, Finland; 20 Department of Transplantation and Liver Surgery, University of Helsinki and Helsinki University Hospital, Helsinki, Finland; 21 Heim Pál National Institute of Pediatrics, Budapest, Hungary; 22 Department of Pathology, University of Szeged, Szeged, Hungary

**Keywords:** Gastroenterology, Gastrointestinal diseases, Pancreatic disease, Pancreatitis

## Abstract

Chronic pancreatitis (CP) is an end-stage disease with no specific therapy; therefore, an early diagnosis is of crucial importance. In this study, data from 1315 and 318 patients were analysed from acute pancreatitis (AP) and CP registries, respectively. The population from the AP registry was divided into AP (n = 983), recurrent AP (RAP, n = 270) and CP (n = 62) groups. The prevalence of CP in combination with AP, RAP2, RAP3, RAP4 and RAP5 + was 0%, 1%, 16%, 50% and 47%, respectively, suggesting that three or more episodes of AP is a strong risk factor for CP. Laboratory, imaging and clinical biomarkers highlighted that patients with RAP3 + do not show a significant difference between RAPs and CP. Data from CP registries showed 98% of patients had at least one AP and the average number of episodes was four. We mimicked the human RAPs in a mouse model and found that three or more episodes of AP cause early chronic-like morphological changes in the pancreas. We concluded that three or more attacks of AP with no morphological changes to the pancreas could be considered as early CP (ECP).The new diagnostic criteria for ECP allow the majority of CP patients to be diagnosed earlier. They can be used in hospitals with no additional costs in healthcare.

## Introduction

Chronic pancreatitis (CP) is a severe condition that greatly deteriorates quality of life and decreases life expectancy. Importantly, there is no specific curative intervention available^1–3^. Patients with CP typically struggle with pain, stigmatization, unemployment and depression^4,5^. The diagnostic criteria for CP only allow us to detect the disease at the end stage, at which around 90% of pancreatic parenchyma is already irreversibly damaged^6^. Another issue is that 68% of cases are caused by alcohol, which negatively influences patients’ compliance with any therapy^7^. Given these facts, it is not surprising that only 20 studies investigating CP are registered in the leading trial registries (ISRCTN and clinicaltrials.gov), a number which is not comparable to the number of studies on pancreatic cancer (114 trials).

One of the possibilities to increase research activity in the field is to diagnose CP earlier. The Japan Pancreatic Society (JPS) has already provided a definition of early CP (ECP). They recommend that patients who fail to qualify for a definitive or probable diagnosis of CP, continuously drink more than 80 g/day alcohol and show at least two out of seven characteristic findings on endoscopic ultrasonography (EUS) should be diagnosed with ECP. However, this diagnosis may be uncertain, since it includes patient- and observer-reported components^8^. The four leading pancreatic societies (IAP, APA, JPS and EPC) have attempted to reach an agreement on the definition of ECP; however, even after long and exhaustive discussions, consensus has not been reached. The board of investigators stated that ECP cannot be diagnosed by any single biomarker, since all early biomarkers are nonspecific^9^.

Several pathomechanisms were proposed earlier to describe disease progression towards CP^10,11^. Sankaran et al. based on their meta-analyzis investigated a frequency of progression from AP to CP. They has found that more than 10% of patients with a first episode of AP and 36% of patients with recurrent AP develop CP^12^. Another model approaching the transition of AP towards CP is the sentinel acute pancreatitis event (SAPE)^13^.

DeSouza et al. reported a high-quality MR study which was first to demonstrate ‚pancreas shrinkage ‘ after ≥ 3 attacks of AP^14^. However, large cohort analyses are still lacking. Theoretically, a high-quality acute pancreatitis (AP) registry, in which the number of attacks is recorded and a large number of variables are collected, may help us to identify biomarkers indicating the early stage of CP. If experimental data confirm significant differences in the levels of these biomarkers in RAP and CP patients, we can propose an improved definition of ECP over the currently available one from the JPS.

## Results

### One out of five patients with an acute episode suffers from RAP, as opposed to one out of twenty who has CP

**Figure 1:**
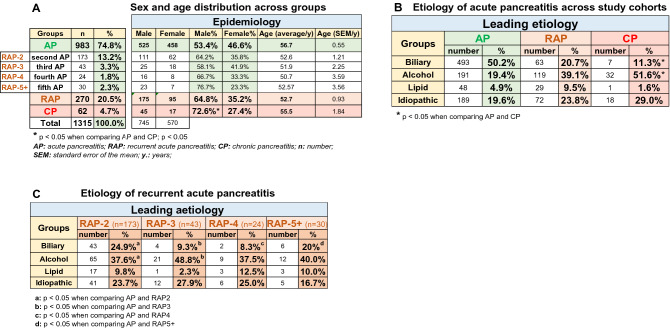
Epidemiology and etiology of pancreatitis in the study population. (**A**) Sex and age distribution across groups. (**B**) Etiology of pancreatitis across study cohorts. (**C**) Etiology of recurrent acute pancreatitis (RAP).

Out of the 1315 patients in the AP registry, a cohort of 983 (74.8%) patients had AP without CP, as opposed to 270 (20.5%) suffering from RAP without CP; 62 (4.7%) of the acute episodes were accompanied by already existing CP (shown in Fig. 1A, for the flowchart of patient selection from the AP registry, see Sup Fig 1). Two-thirds of RAP cases had fewer than three attacks, as opposed to one-third experiencing three or more attacks. As regards sex and age, there is a clear trend in the groups. The male/female ratio was 53.4/46.6% with AP, 64.8/35.2% with RAP, as opposed to 72.6/27.4% with CP. The average age at the first attack in patients with AP was 56.7 ± 0.55, that of RAP was 52.7 ± 0.93, and that of CP was 55.5 ± 1.84 (Fig. 1A).

### Bidirectional changes in alcoholic and biliary etiologies in AP, RAP and CP patients

As regards etiology, 50.2% of AP cases were biliary; however, the rate of this etiological factor continuously decreased towards CP (20.7% with RAP and 11.3% with CP). The distribution of alcoholic etiology moved in the opposite direction: 19.4% with AP, 39.1% with RAP and 51.6% in the CP group (Fig. 1B). The increasing proportion of cases with an unknown etiology deteriorating from AP (19.6%) to CP (29%) could either be due to a higher rate of genetic predisposition^15^ or the attrition of alcohol-dependent patients (Fig. 1B). Leading etiologies in RAP are shown in Fig. 1C.

### Local complications are more frequent in CP than in AP or RAP, whereas systemic complications and mortality are less so

**Figure 2:**
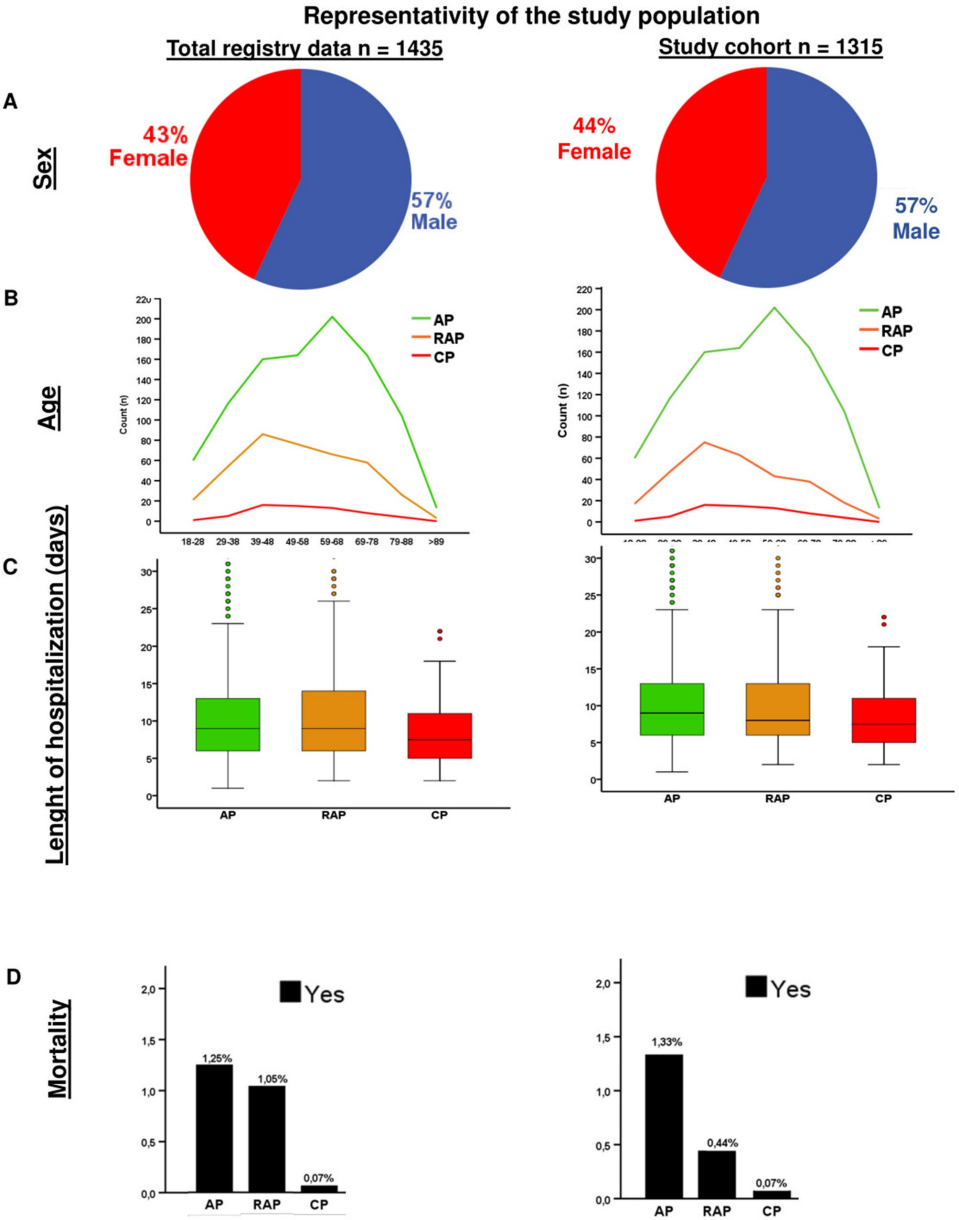
Representativeness of the study population. (**A**) Sex distribution. (**B**) Age distribution. (**C**) Lengths of hospitalization. (**D**) Mortality.

The demographic, epidemiological and primary outcome parameters suggest that after each acute episode of AP, patients move closer to CP. Therefore, investigating all biomarkers collected from patients during episodes of AP, irrespective of its relationship with the pancreas, may help us to recognise ECP. Thus, in the next part of the study, we systematically analysed all the 102 biomarkers (data quality is shown in Sup Table 1). The primary outcomes of analysed data are shown in Fig. 2.

### Fifteen out of 102 biomarkers showed significant alterations between the AP, RAP and CP groups

**Figure 3:**
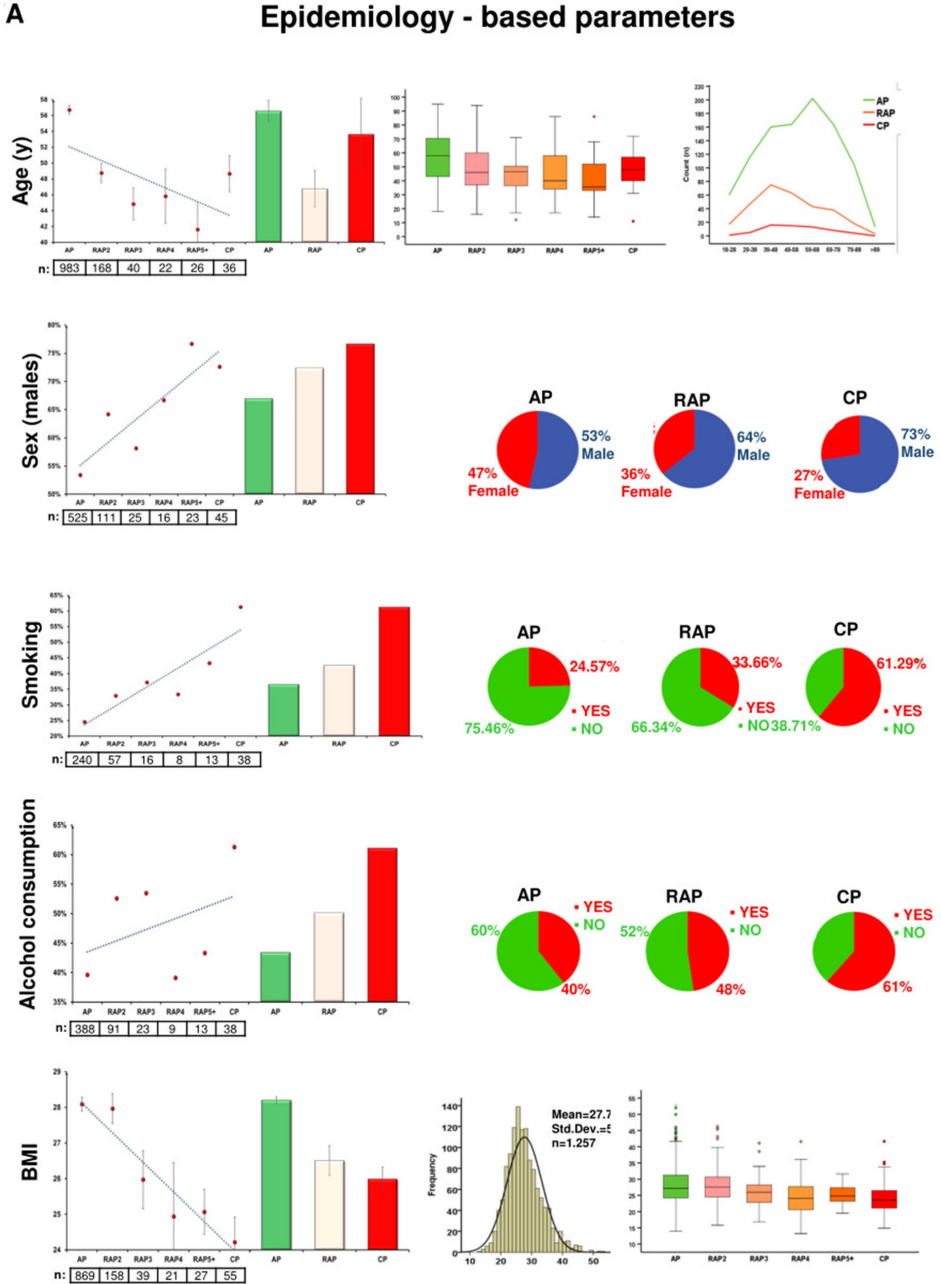

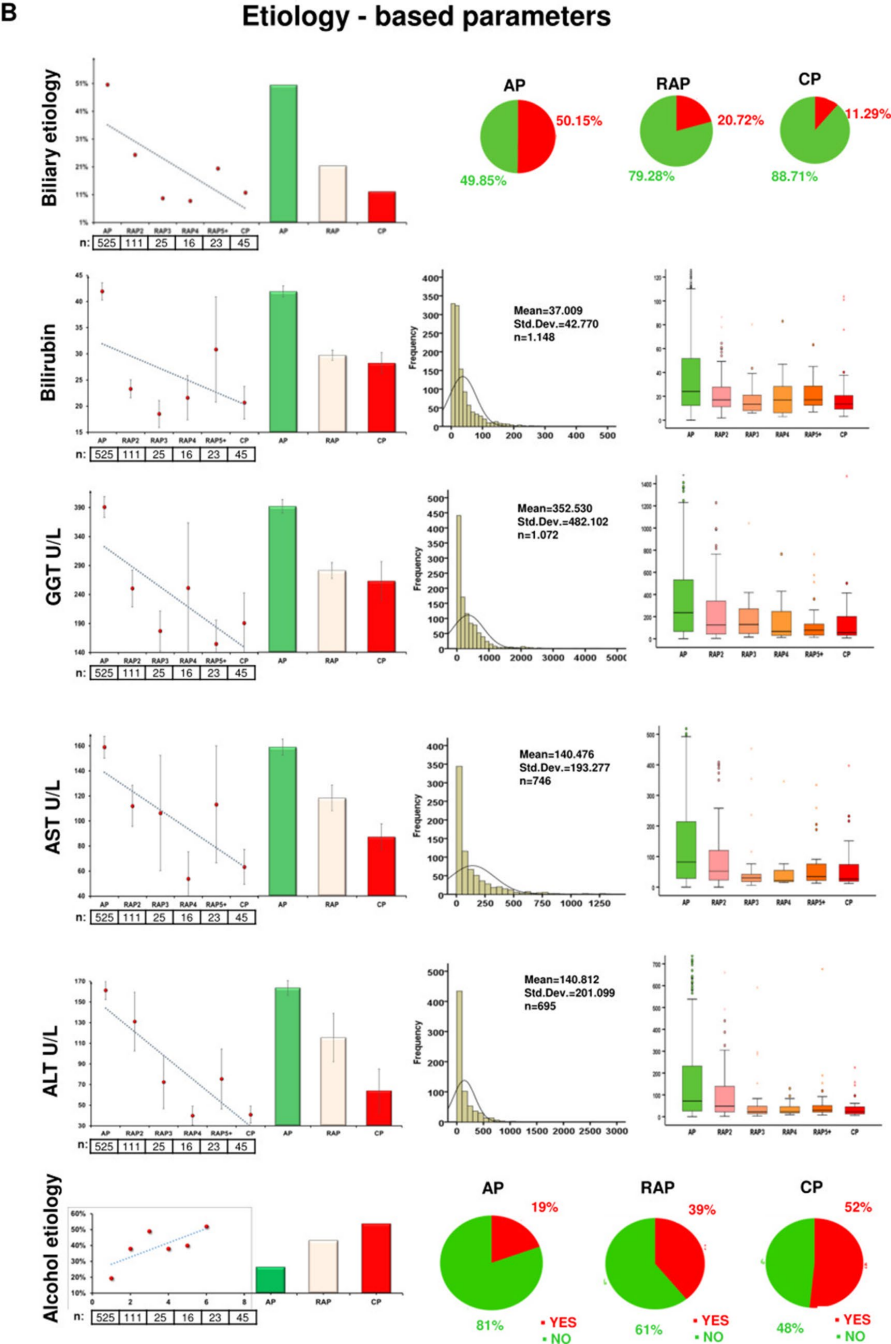

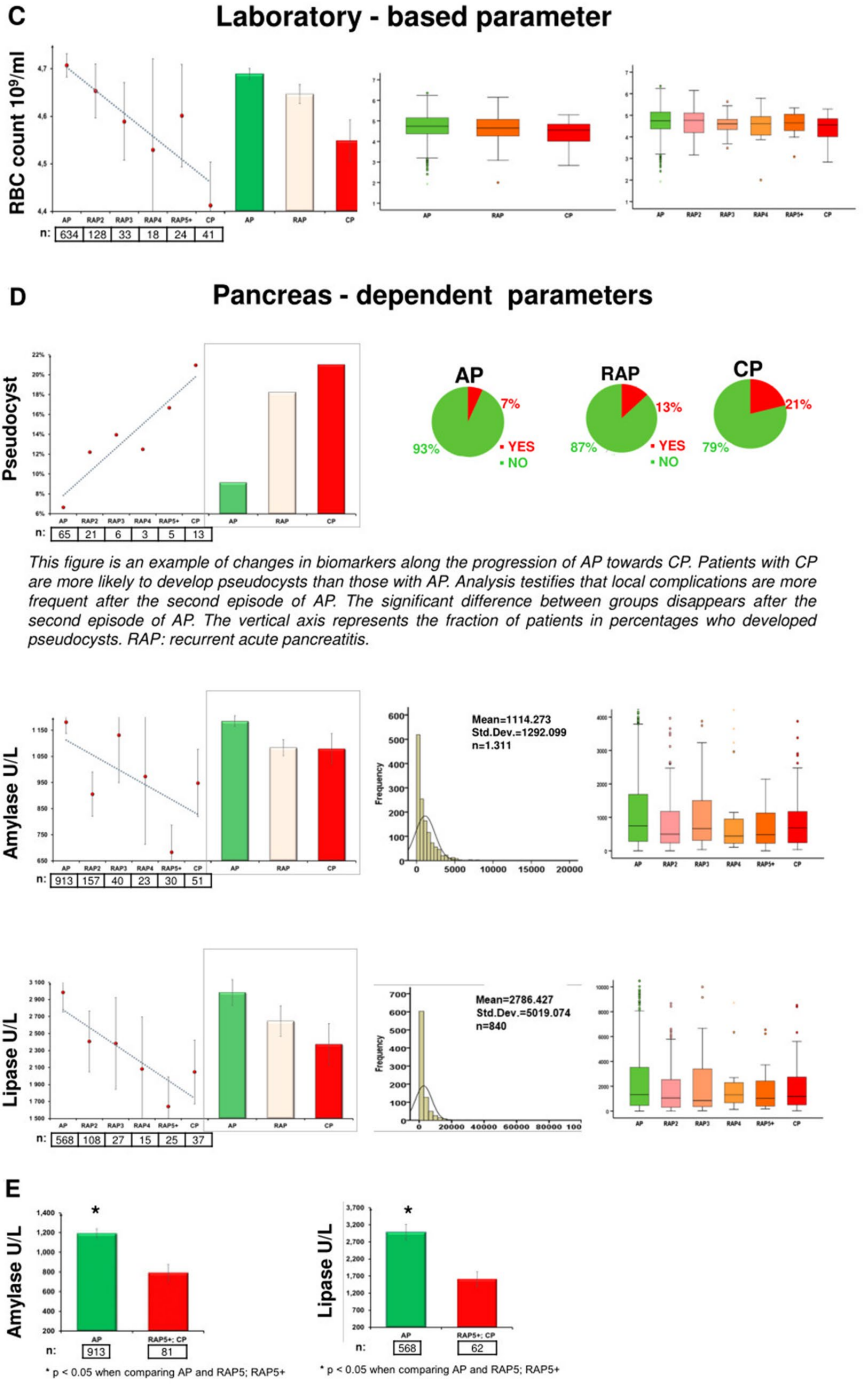
Descriptive and comparative statistics characterising fifteen biomarkers. (**A**) Epidemiology-based parameters: age, sex, smoking, alcoholic etiology and body mass index (BMI). (**B**) Etiology-based parameters: biliary or alcoholic etiology, bilirubin, gamma-glutamyl transferase (GGT), aspartate aminotransferase (AST) and alanine aminotransferase (ALT). (**C**) Laboratory-based parameter: red blood cell count (RBC). (**D**) Pancreas-dependent parameters: pseudocysts, amylase and lipase. (**E**) Differences in acute pancreatitis (AP) vs recurrent acute pancreatitis (RAP) 5 + and chronic pancreatitis (CP) comparisons in amylase and lipase.

Figure 3 shows the descriptive and comparative statistics of the fifteen biomarkers investigated. Five of them were epidemiology-based (age, sex, smoking, alcohol consumption and body mass index (BMI)) (Fig. 3A), six were etiology-based (biliary and alcoholic etiology, serum levels of bilirubin, gamma-glutamyltransferase (GGT), aspartate aminotransferase (AST) and alanine aminotransferase (ALT)) (Fig. 3B), one was a laboratory parameter (red blood cell count (RBC)) (Fig. 3C), and three were pancreas-dependent parameters (rate of pseudocyst formation, serum amylase and lipase) (Fig. 3D). In 13 biomarkers, there were significant differences between the groups (Fig. 3A–D). In two parameters (serum lipase and amylase), due to the distorting effect of the low *n* number in the CP group, no significant differences were detected between the AP and CP groups. However, when we elevated the *n* numbers in the CP group by merging the CP and RAP5 + groups (as the latter is biologically very close to the CP group), the statistical difference could be detected (Fig. 3E). All in all, the dynamic changes of these parameters indicate that RAP represents a continuous transition from AP to CP.

### The significant differences between the biomarkers measured during acute episodes in AP and CP disappear after 2–3 attacks

**Figure 4:**
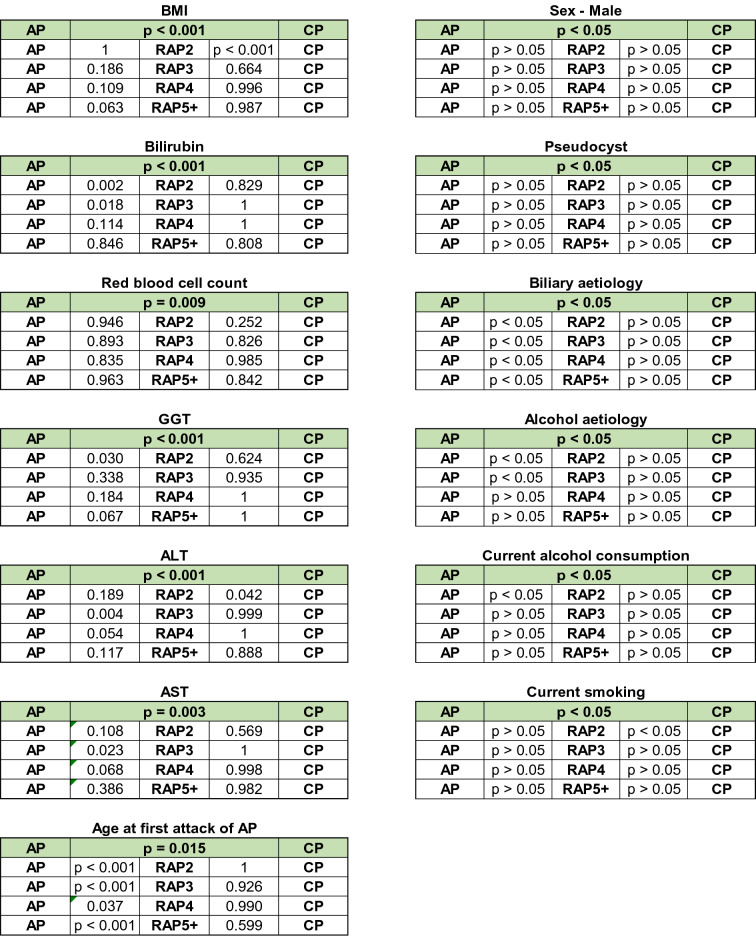
Significant differences between thirteen biomarkers.

In these series of analyses, we aimed to determine the number of episodes of AP (without pancreatic morphological changes) required for significant differences in biomarkers to vanish compared to CP. The significant difference in eleven parameters disappears after the second episode of AP (RAP2; age, sex, etiology, alcohol consumption, bilirubin, AST, GGT, RBC count, pseudocysts, amylase and lipase). The significant difference in BMI and ALT disappears after the third episode of AP (RAP3). There is no difference in smoking after the fourth episode of AP (RAP4) (Fig. 4). Calculating the rate of morphological alterations after each episode revealed that 0.3% of AP, 1% of RAP2, 16% of RAP3, 33% of RAP4 and 32% of RAP5 + cases already have either CT-, MRI-, US- or EUS-based morphological alterations.

These data indicate that there is a stage in CP development in which biomarkers show the disease progression earlier than pancreatic morphological changes. Characteristics of patients with AP (non-ECP and non-CP), ECP (non-CP) and CP are summarized in Sup Table 2.

### In the RAP3 group, 16% of patients already have established CP, while the figure is nearly 50% in the RAP4 + group

**Figure 5:**
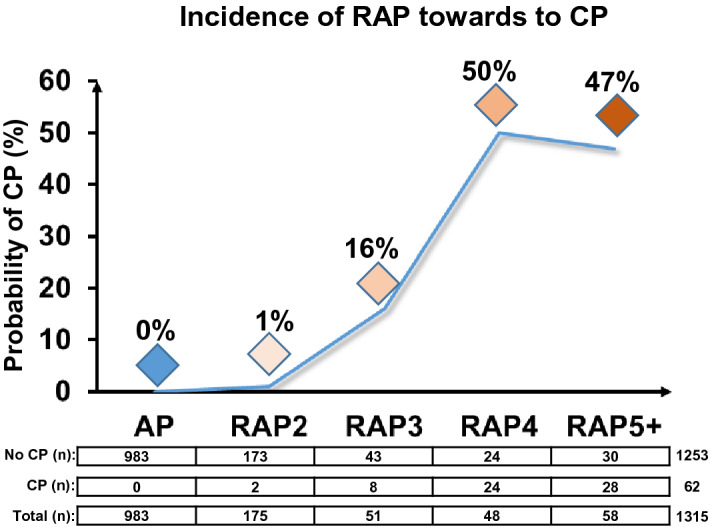
Progression from the first acute pancreatitis (AP) episode through recurrent episodes (RAP) towards chronic pancreatitis (CP).

We also investigated whether the incidence of recurrent episodes increases the chance of CP development. Patients having had one or two episodes of acute inflammation had negligible odds of developing CP (less than 1%); however, patients who had experienced three episodes had a 16% chance of developing CP, and patients with four or more episodes had a 50% likelihood. These data demonstrate that three or more episodes of AP are to be considered as a significant risk factor for the development of CP (Fig. 5).

### RAP patients have an average of three attacks, whereas CP patients have an average of four to five

RAP patients had an average of 3.07 ± 1.85 AP attacks at the time of diagnosis, whereas CP patients had 3.76 ± 2.24 in the AP registry. We continued our analysis of data from the CP registry, which includes 366 patients. Out of this population, 324 cases had data on the number of attacks at the time of enrolment in the CP registry (for the flowchart of patient selection from the CP registry, see Sup Fig. 2). In CP, 318 out of 324 patients had at least one acute episode: 69 CP patients had one acute episode (21.6%), 66 had two (20.8%), 66 had three (20.8%), 29 had four (9.1%), and 88 had five or more (27.7%). The average number of attacks was 4–5 (4.07 ± 3.82).

### Experimental data confirm that three or more episodes of AP cause a significant decrease in serum amylase levels and induce CP-like morphological changes

**Figure 6:**
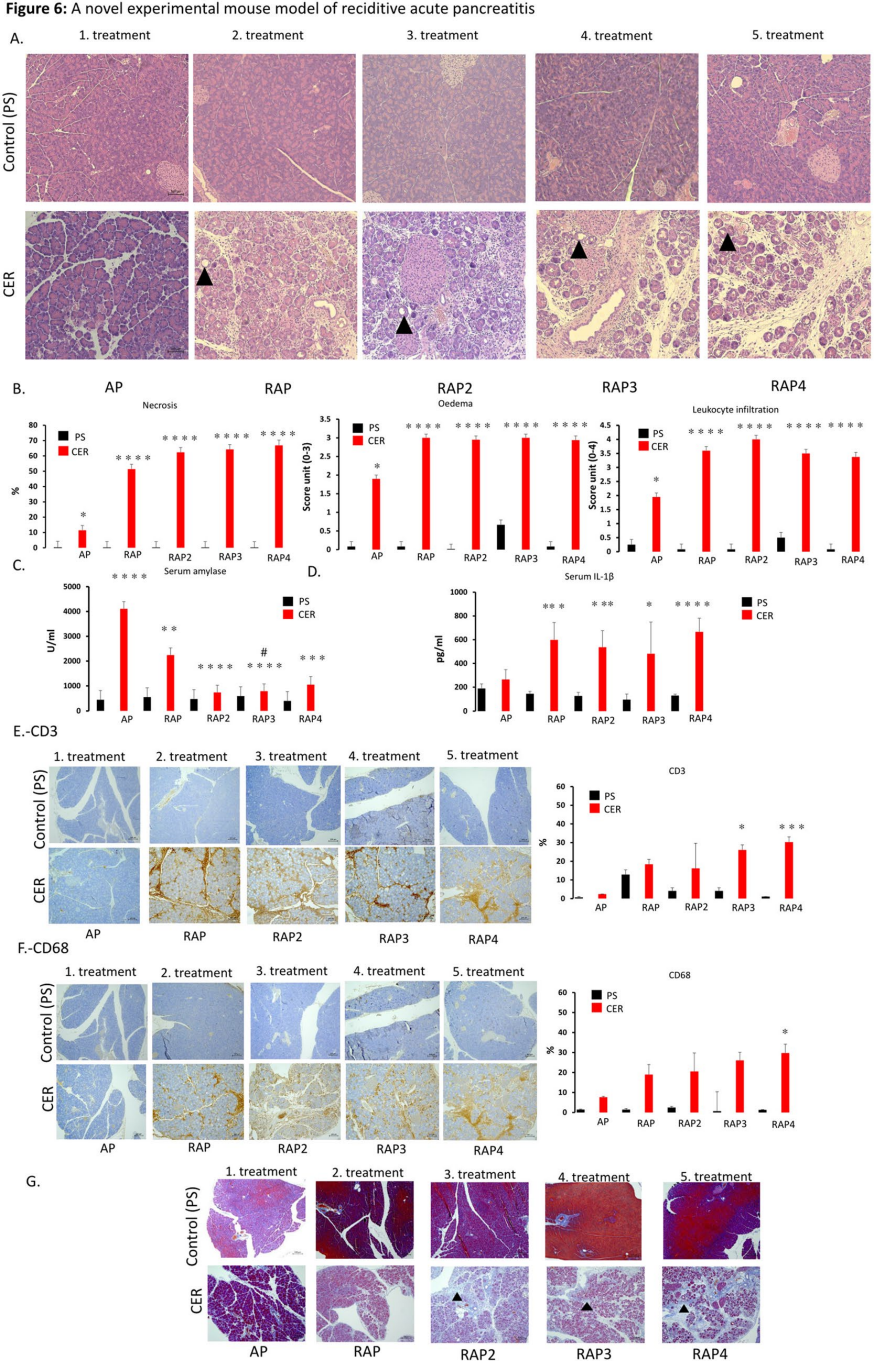
A novel experimental mouse model of reciditive acute pancreatitis and chronic pancreatitis. (**A**) Histological findings in an experimental mouse model of acute pancreatitis. (**B**) Necrosis, edema and leukocyte infiltration differences in pancreatitis stages in an experimental mouse model. (**C**) Serum amylase activity and (**D**) Serum IL-1β level differences in stages of pancreatitis in an experimental mouse model. (**E**) CD3 (T cell, brown colour) staining. (**F**) CD68 (macrophage, brown colour) staining. (**G**) Fibrosis (blue colour filled triangle), each scale bar represents 100 µm. * = *p* < 0.05. A detailed description can be found in Supplementary Document 1.

In our clinical dataset, on-admission pancreatic enzyme levels continuously decreased from AP to CP. However, we had two problems with this dataset. Firstly, it only showed a significant difference when CP was merged with RAP5 + (due to the low *n* number, see above). Secondly, our database did not provide clinical evidence on whether this continuous decrease is linked to the loss of pancreatic parenchyma. Therefore, we established a preclinical model of RAP (and used a novel dosing schedule to induce pancreatitis) to investigate the effect of recurrent inflammation on on-admission amylase activity, pro-inflammatory cytokine level (measuring IL-1ß), inflammatory cells (macrophage and T cells) and pancreatic morphology. We found that repeated AP inductions (Sup Fig 3) can mimic histological changes from RAP to CP. We found strong evidence that repeated AP induction leads to severe fibrosis, necrosis, leukocyte infiltration, including T cells and macrophages, elevation of pro-inflammatory cytokine level and edema of the pancreas (Fig. 6A–G). Serum amylase activity (Fig. 6C) was highly elevated in the case of AP. After that, each episode reduced the rate of increase in serum amylase activity. For details, please see Supplementary Document 1. Our data show that, after the second episode of AP, there is a significant drop in serum levels of amylase in each concomitant episode of AP, which is associated with pancreatic parenchymal damage (i.e. episode-dependent acinar depletion).

## Discussion

There is a large amount of data available on AP and CP; however, much less is known about RAP and ECP. Therefore, it is not surprising that clinicians of the four major pancreatic associations failed to reach an agreement on diagnostic criteria for ECP. The only association that has attempted to describe ECP is the Japan Pancreas Society. However, their guidelines are complicated, with only a limited possibility for use in general practice^8,16^. Two nationwide studies have already highlighted that repetitive inflammation of the pancreas can lead to CP^17,18^. In a cross-sectional epidemiological study, Masamune et al. showed that 26.5% of ECP cases had previous acute episodes^17,18^. Importantly, the incidence rate of RAP was much higher in a two-year prospective follow-up study, in which 75% of patients had AP before ECP was diagnosed. Cho et al. in their nationwide cohort study investigated the influence of cholecystectomy and RAP events on the risk of post-pancreatitis diabetes mellitus. Patients who had 2 or ≥ 3 recurrent biliary events prior to cholecystectomy were at a significantly increased risk of post-pancreatitis diabetes mellitus. The results of your study are in line with that study^19^. These data support the SAPE model describing the transition of AP towards CP. According to this model, the first (so-called sentinel) episode of acute pancreatitis (SAPE) triggers a cellular activation cascade, which leads to chronic pancreatic inflammation and fibrosis. The model proposes a tipping point in time when, in line with the multihit theory model, the effects of risk factors, such as alcohol consumption and smoking, turn into etiological factors, triggering the cascade that ends up in CP^20^. Findings from a cohort study by Sheel et al. were in line with this model^13^. In our international cohort, we investigated uniformly and prospectively collected 130,744 pieces of high-quality data from 1315 patients. Our epidemiological analysis revealed that one out of five AP patients suffers from RAP, whereas one out of twenty suffers from CP, which data were reported by the Japanese cohort studies. Of note, almost all the CP cases (98%) had a previous episode of AP, which is surprisingly high compared to previous data by Olesen et al. (47%)^21^.

We found 15 variables that were significantly different in the first AP and CP, and, importantly, the differences start disappearing after recurrent episodes of AP. Epidemiological data showed that the male gender, younger age and lower BMI are associated with RAP and CP, which data are in accordance with the findings of the Cleveland cohort, where the average age of the first AP was 55.5 ± 16.6y, that of the second was 53.8 ± 18.5y, and that of the third 45.2 ± 12.4y. Importantly, no further changes were observed after the third attacks of AP (45.7 ± 16.5y), suggesting that three or more AP attacks may be a separate group of RAP^22^.

One of the key findings of this study is that the incidence of recurrent episodes increases the risk of CP development. The first two attacks have small effects (0–1%) on the odds for developing CP, whereas the third and fourth (16–50%) episodes have large ones. The striking difference between RAP2 and RAP3 could be explained by at least three factors: (1) The biliary etiology decreased from 24.9 to 9.3%, whereas the alcoholic etiology elevated from 37.6 to 48.8%. While the biliary etiology is usually a one-time hit on the pancreas, alcohol has a continuous deteriorating effect. (2) RAP3 occurs in a more damaged pancreas, which is actually confirmed by our experimental settings. (3) RAP3 seems to be more severe than RAP2 (mortality: 4.7% vs 2.3%; systemic complications: 4.7% vs 2.3%).

In a longitudinal study by Lankisch et al.^23^ from Germany, CP was diagnosed after the second RAP in nine patients, after the third RAP in seven and after the fourth in three. Heavy smoking (> 30 cigarettes per day) predisposed patients to CP after the first AP attack. The role of smoking was also confirmed by the findings of Yadav et al.^24^ Importantly, they found that the strongest predictor for a subsequent diagnosis of CP was RAP (HR = 4.57, 95% CI: 3.40–6.14).

Our significant biomarkers clearly showed bidirectional changes in alcoholic and biliary etiologies in AP, RAP and CP patients. Similar changes were found in the Cleveland^22^, Chinese^25^ and Central European^1^ cohorts as well. These data suggest the usefulness of cholecystectomy in AP. Before the routine cholecystectomy era, biliary AP was even more frequent in RAP than in general AP^26^. The fact that the biliary etiology in CP (11.29%) is five times less than in the first episode of AP (50.15%) also suggests that repetitive episodes are one of the key determinants of CP. Importantly, Bertilssom et al.^27^ from Denmark reported that one or more AP episodes are among the strongest predictors for the development of CP.

The linear changes in the values of pancreas-dependent parameters (rate of pseudocysts, serum amylase and lipase) suggest that repetitive attacks lead to local damage of the pancreas, which is one of the hallmarks of CP. The elevation of local complication rates was reported in earlier published cohorts as well^22,25,27–30^.

We report that the on-admission elevation of serum lipase and amylase levels decrease from AP to CP via RAP. One of the most likely explanations is that these changes are due to the loss of pancreatic acinar cells. Therefore, since histological samples were not available from the members of our cohort, we investigated this hypothesis in an experimental CP model. As with human observations, the serum amylase level continuously decreased after the second and third attacks, which changes were associated with the loss of pancreatic parenchyma and enhanced fibrosis.

DeSouza et al. in their firstly reported high-quality MR study demostrated ‚pancreas shrinkage ‘ after ≥ 3 attacks of AP^14^. A total of 123 participants were studied. Total pancreas volume (TPV) and tail diameter were significantly reduced in both unadjusted (TPV (*p* = 0.036), tail diameter (*p* = 0.009)) and adjusted (TPV (*p* = 0.026), tail diameter (*p* = 0.034)) models in individuals with ≥ 3 attacks, but not with 1 or 2 attacks, compared with healthy individuals. These results are strongly correlated with our results.

The episode-dependent decrease in the elevation of amylase activity highlights the loss of acinar cells in the pancreas and the functional decrease of pancreatic enzyme secretion/leakage from acinar cells. Importantly, these data also indicate that the three-fold elevation of serum pancreatic enzymes may not be suitable for setting up a diagnosis in patients suffering from RAP. Taken together, both the clinical and experimental studies suggest that three or more episodes of RAP can be considered as ECP.

One of the major limitations of the study is the cross-sectional design: the patients were not followed longitudinally from AP through RAP to CP. Therefore, we have no information on the time of diagnosis of CP and the time relationships of RAP and CP. We decreased the limitations by analysing our CP cohort as well, in which patients were collected separately from our AP cohort. It is also important to note that some of the patients suffering from CP had no reported AP before their CP diagnosis. Therefore, our definition of ECP does not cover all ECP patients. Most cases of CP were confirmed with abdominal US and CT, which modalities have less diagnostic specificity and sensitivity for CP than EUS or MRCP. When abdominal US and CT showed Wirsung dilatation or calcification and the etiology was known (usually with a background of alcoholism), investigators did not use EUS or MRCP. Therefore, more detailed analysis of the results of EUS or MRCP were not possible in this study. Our patient registries record only routine laboratory parameters, while analysing cytokine levels may have added extra information on the pathogenesis of transition. Statistical limitations include the fact that, since our publication has a hypothesis-generating purpose, we did not adjust tests for multiplicity (except in the ANOVA model). In addition, data quantity and quality did not allow us to perform multivariate statistics. Mild differences in management strategies across centres may affect disease outcomes. To decrease the limitations described here, we started a new international, observational, longitudinal investigation of acute pancreatitis, entitled the GOULASH PLUS study, in which we will monitor all AP patients for six years to characterise ECP more precisely^31^.

In addition to the limitations noted above, our research has several highly important advantages: (1) it can be used easily in all hospitals, (2) no additional laboratory measurements or imaging techniques are necessary after ruling out CP with imaging to establish a diagnosis of ECP, and (3) it allows us to start clinical trials and encourage patients to implement lifestyle changes to prevent the development of CP from ECP. Our results adds to the accumulating body of morphological^14^ and population-based^19^ studies on this topic. Our study shows that three or more episodes of RAP with no pancreatic morphological alterations may be considered ECP. Results from validation studies, such as the GOULASH PLUS study, are still forthcoming.

## Materials and methods

### The clinical part of the study

#### Design

This study is a comparative cross-sectional study. The study is being reported according to the STROBE Statement.

The Hungarian Pancreatic Study Group (HPSG) developed international registries for pancreatic diseases (AP, CP and pancreatic cancer)^3,28,32^. All pancreatic centres worldwide can join these registries (https://tm-centre.org/en/registries/gastroenterology-en/). In this study, we extracted and analysed data from the AP and CP registries.

The AP registry contained data from 1435 patients, 1315 of whom from 28 centres in twelve countries collected between June 2012 and September 2017 were eligible for analysis. Centre distribution is displayed in Sup Fig 4, whereas patients eligible for inclusion can be found in Sup Fig 1.

One hundred and two individual variables (on-admission biomarkers, defined as laboratory test and imaging results, demographic factors or any relevant information from the medical history) were found to be eligible for investigation (Sup Table 1).

The CP registry contained clinical data from a total of 366 patients’ from 25 centres in two countries collected between June 2012 and September 2017, 324 of whom (88.5%) were eligible for inclusion (Sup Fig 2). Centre distribution is displayed in Sup Fig 5. No additional biomarkers from the CP registry were investigated.

#### Definition of pancreatitis

Diagnosis of AP was established based on the recommendations of the HPSG guideline (adapted and updated from APA/IAP guideline)^33^, while that of CP was based on the HaPanEU criteria^1,34,35^.

#### Patient groups

We formed three groups based on the morphology of the pancreas and the number of recurrent acute episodes. The first episode of AP without any chronic morphological change of the pancreas was labelled AP (983 cases). Cases with two or more episodes of AP without clinical signs and symptoms of CP or pancreatic morphological alterations were labelled recurrent acute pancreatitis (RAP) (270 cases). RAP was further divided into four subgroups based on the number of acute episodes (RAP2: two episodes of AP; RAP3: three episodes; RAP4: four episodes; and RAP5 + : five or more episodes). RAP 5 + consisted of 30 cases: twelve cases with five acute episodes, four cases with six, six cases with seven, three cases with eight, two cases with ten, two cases with eleven, and one case with twelve. Any acute episodes based on clinical signs and symptoms and pancreatic morphological changes attributed to chronic inflammation were labelled CP (62 cases).

#### Ethical approval for the clinical study

The study was approved by the Scientific and Research Ethics Committee of the Medical Research Council (22,254–1/2012/EKU). All participants in this study provided written, informed consent. Use of data from the AP and CP registries was permitted by the Hungarian Pancreatic Study Group (HPSG).

### The experimental part of the study

#### Animals

A total of 40 wild-type (WT) FVB/N mice were sacrificed. Mice (male, aged between 20 and 25 days) were housed in a room maintained at a temperature of 20–22 °C on a 12 h light–dark cycle with food and water available.

#### Experimental model

Although there are some protocols available for inducing chronic pancreatic damage in mice with caerulein (CER)^36,37^, we introduced a novel experimental methodology that can be used to study the differences and alterations in different phases of pancreatic injury. 50 µg/kg CER was administered i.p. ten times within ten hours to induce AP on Day 1. This method was repeated to induce RAP on Day 4, to induce ECP on Days 4 and 7 and to induce CP on Days 4, 7, 10 and 13^38,39^. Buprenorphine with a concentration of 0.1 mg/kg s.c. was administered to the mice every six hours each day of the treatment to relieve pain. The mice were euthanized with 200 mg/bwkg pentobarbital i.p. (Bimeda MTC, Cambridge, Ontario, Canada) two hours after the last injections of CER. A cardiac puncture was performed on the mice for exsanguination, and the pancreas glands were removed. Blood samples were collected from the cardiac puncture and placed on ice immediately. These samples were centrifuged at 2500 RCF for 15 min at 4 °C, and blood serum was collected and stored at -20 °C until analysis. Pancreas samples were placed into an 8% formaldehyde solution and stored at -4 °C. The formaldehyde-fixed pancreas samples were embedded in paraffin and cut into 3 μm thick sections and stained for Crossman’s Trichrome staining or hematoxylin–eosin using a standard laboratory method (Department of Pathology, University of Szeged, Szeged, Hungary). Immunohistochemistry was performed (Department of Pathology, University of Szeged, Szeged, Hungary) to detect CD3 (MRQ-39, Rabbit Monoclonal Antibody, CellMarque, CA, USA) and CD68 (Anti-Macrosialin CD68 Antibody, Booster Bio, CA, USA) in the pancreas samples. Image J Software was used to evaluate the positive stainings for CD3 and CD68. A Mouse IL-1β ELISA Kit (EM0109, Wuhan Fine Biotech Co, China) was employed to measure IL-1β levels in serum samples, with absorbance of the samples detected at 405 nm. A semi-quantitative scoring procedure was performed to quantify the edema, necrosis and leucocyte infiltration levels of the hematoxylin–eosin stained slides^40,41^. A colorimetric kit was used (Diagnosticum, Budapest, Hungary) to measure serum amylase activity, with absorbance of the samples detected at 405 nm with a FLUOstar OPTIMA (BMG Labtech, Germany) plate reader.

#### Ethical approval for the experimental study

Local ethical committees approved experiments on investigations involving animals at the University of Szeged (XII/4988/2015). The experiments were performed in compliance with European Union Directive 2010/63/EU and Hungarian Government Decree 40/2013 (II.14.).

### Statistical analyses

The mean ± standard error of the mean (SEM) values were calculated for descriptive statistics. The numbers and percentages of cases were computed for categorical variables. To check the normality of the data, we used the Kolgomorov-Smirnov test and/or visual inspection of the Q–Q plots. In the case of non-normal distributed variables, e.g., bilirubin, GGT, AST, ALT, amylase and lipase, we transformed our data into the logarithmic scale to achieve normal distribution; then we applied parametric tests (graphs were prepared from the raw data without transformation). To identify significant differences between groups, we used the following statistical tests for the whole dataset and subgroups as well.

To observe differences between two groups, the independent t-test was applied (Figs. 1A, 3E, 6), to compare more than two groups; we used one-way ANOVA with a Tukey post hoc test to adjust the results for alpha error (Figs. 3A–D, 4). The association between categorical variables was examined with the Chi-square test and the Fisher's exact test, depending on the sample size (Figs. 1, 3A,B,D). All statistical tests were performed using SPSS statistical software version 25 (IBM Corporation, Armonk, NY).

## Supplementary Information

Supplementary Information.
