# Supplementary material for: Evidence for diagnosis of early chronic pancreatitis after three episodes of acute pancreatitis: a cross-sectional multicentre international study with experimental animal model
Source: Sci Rep. 2021 Jan 14;11:1367. doi: 10.1038/s41598-020-80532-6 (PMC7809468; doi:10.1038/s41598-020-80532-6)
Supplement: Supplementary file 1 — Supplementary Information. [file 41598_2020_80532_MOESM1_ESM.pdf]

Title:

Evidence for diagnosis of early chronic pancreatitis after three episodes of acute pancreatitis: A cross-sectional multicentre international study

Authors:

Péter J. Hegyi,[1,2,3] Alexandra Soós,[4] Emese Tóth,[4] Attila Ébert,[5] Viktória Venglovecz,[5] Katalin Márta,[1] Péter Mátrai,[1] Alexandra K. Mikó,[1,2] Judit Bajor,[2] Patrícia Sarlós,[2] Áron Vincze,[2] Adrienn Halász,[6] Ferenc Izbéki,[6] Zoltán Szepes,[4] László Czakó,[4] György Kovács,[7] Mária Papp,[7] Zsolt Dubravcsik,[8] Márta Varga,[9] József Hamvas,[10] Balázs C. Németh,[4] Melania Macarie,[11] Ali Tüzün Ince,[12] Dmitry Bordin,[13,14,15] Elena A. Dubtsova,[13,14,15] Mariya A. Kiryukova,[13,14,15] Igor E. Khatkov,[15,16] Tanya Bideeva,[17] Artautas Mickevicius,[18] Elena Ramírez-Maldonado,[19] Ville Sallinen,[20,21] Bálint Erőss,[1,2] Dániel Pécsi,[1] Andrea Szentesi,[1,4] Andrea Párniczky,[1,22] László Tiszlavicz,[23] Péter Hegyi[1,2,4,*]

Affiliations:

1 Institute for Translational Medicine, Medical School, Szentágothai Research Centre, University of Pécs, Pécs, Hungary

2 Division of Gastroenterology, First Department of Medicine, Medical School, University of Pécs, Pécs, Hungary

3 Department of Gastroenterology Slovak Medical University in Bratislava, Bratislava, Slovakia

4 First Department of Medicine, University of Szeged, Szeged, Hungary

5 Department of Pharmacology and Pharmacotherapy, Szeged

6 Szent György Teaching Hospital of County Fejér, Székesfehérvár, Hungary

7 Department of Internal Medicine, Division of Gastroenterology, University of Debrecen, Debrecen, Hungary

8 Bács-Kiskun County Hospital, Kecskemét, Hungary

9 Dr. Réthy Pál Hospital, Békéscsaba, Hungary

10 Peterfy Hospital and Trauma, Trauma Emergency Room, Hungary

11 County Emergency Clinical Hospital - Gastroenterology and University of Medicine, Pharmacy, Sciences and Technology, Targu Mures, Romania

12 Hospital of Bezmialem Vakif University, School of Medicine, Istanbul, Turkey

13 A.S. Loginov Moscow Clinical Scientific Center, Moscow, Russia

14 Tver State Medical University, Tver, Russia

15 A.I. Yevdokimov Moscow State University of Medicine and Dentistry, Moscow, Russia

16 A. S. Loginov Moscow Clinical Scientific Center, Moscow, Russia
17 Semashko Central Clinical Hospital, Moscow, Russia
18 Clinic of Gastroenterology, Nephrourology and Abdominal Surgery, Faculty of Medicine, Vilnius University, Lithuania
19 Consorci Sanitari del Garraf, Sant Pere de Ribes, Barcelona, Spain
20 Department of Abdominal Surgery, University of Helsinki and Helsinki University Hospital, Helsinki, Finland
21 Department of Transplantation and Liver Surgery, University of Helsinki and Helsinki University Hospital, Helsinki, Finland
22 Heim Pál National Institute of Pediatrics, Budapest, Hungary
23 Department of Pathology, University of Szeged, Szeged, Hungary
* Email address: hegyi.peter@pte.hu, p.hegyi@tm-centre.org

Supplementary Figure 1: Flow chart

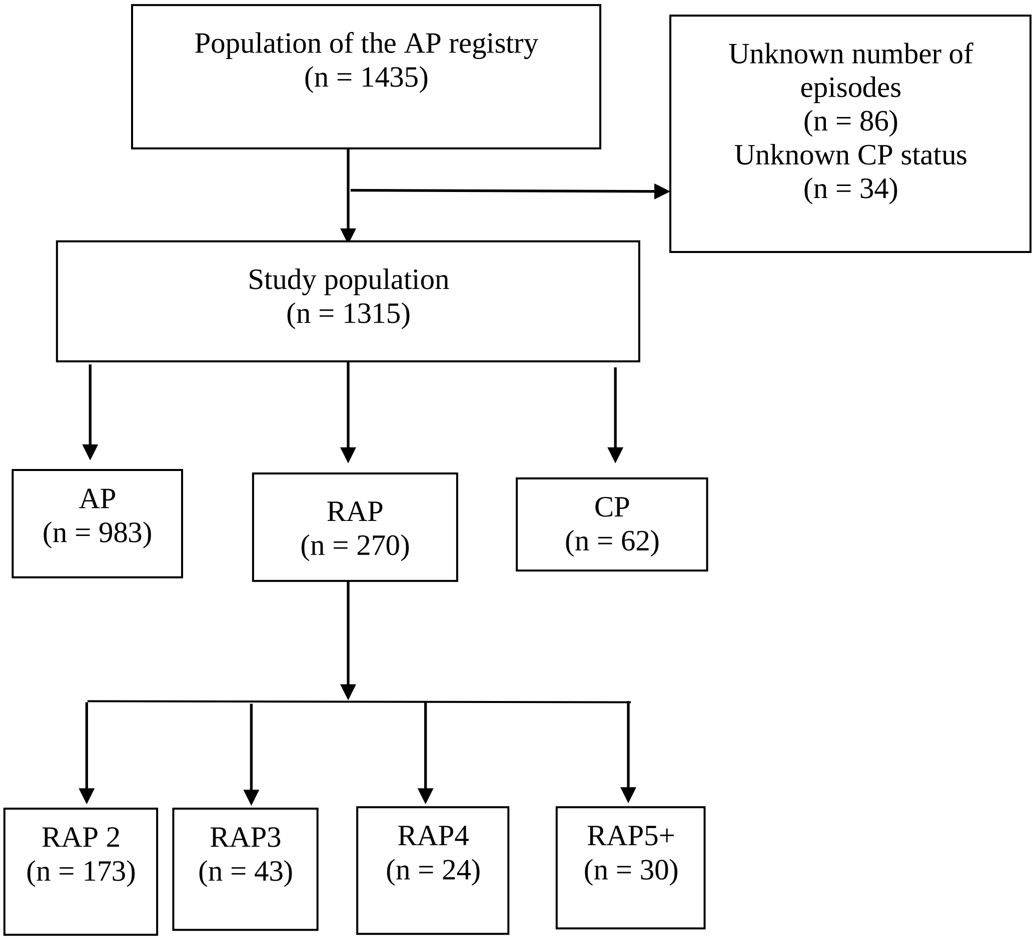

AP: *acute pancreatitis;* **RAP:** *recurrent acute pancreatitis;* **n** = *number of patients*

Supplementary Figure 2: Flow chart

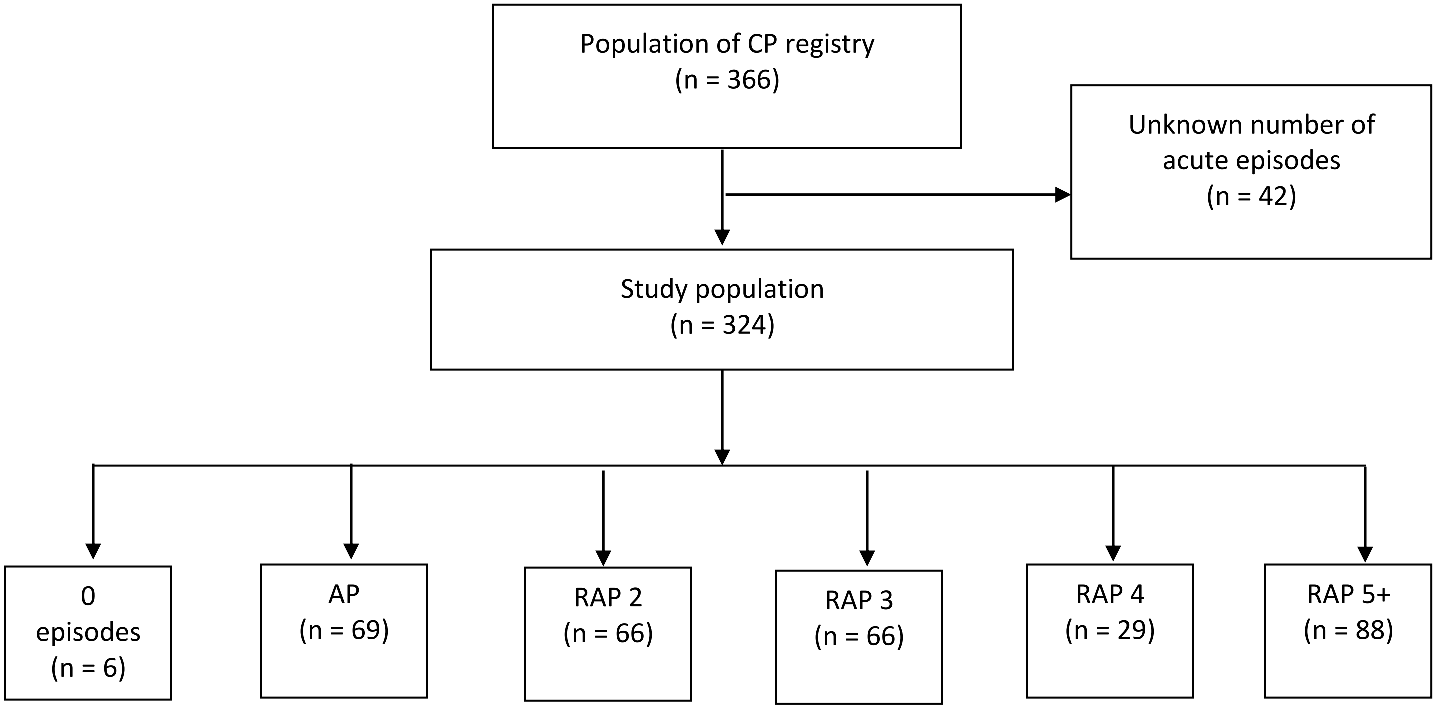

AP: acute pancreatitis (one acute episode in the history); **RAP:** recurrent acute pancreatitis (multiple acute episodes in the history); **n** = number of patients

Spp. Fig.3

The experimental study

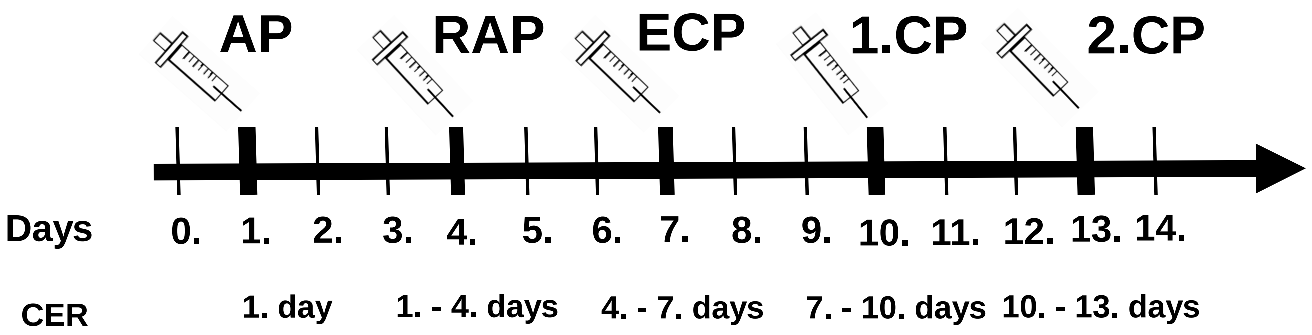

AP; RAP; ECP and CP were induced by CER on a 15 days timescale. CER was injected on every 4th day by hourly dosage of i.p. 50 µg/kg CER 10 times. Each treatment day two groups of animals were sacrified (a group treated with PS and another treated with CER), blood and pancreas samples were collected.

AP: *acute pancreatitis;* ***CER:*** *caerulein;* ***CP:*** *chronic pancreatitis;* ***PS:*** *physiological solution;* ***RAP:*** *recurrent acute pancreatitis;*

Spp. Fig.4 Centre distribution of cases with acute pancreatitis

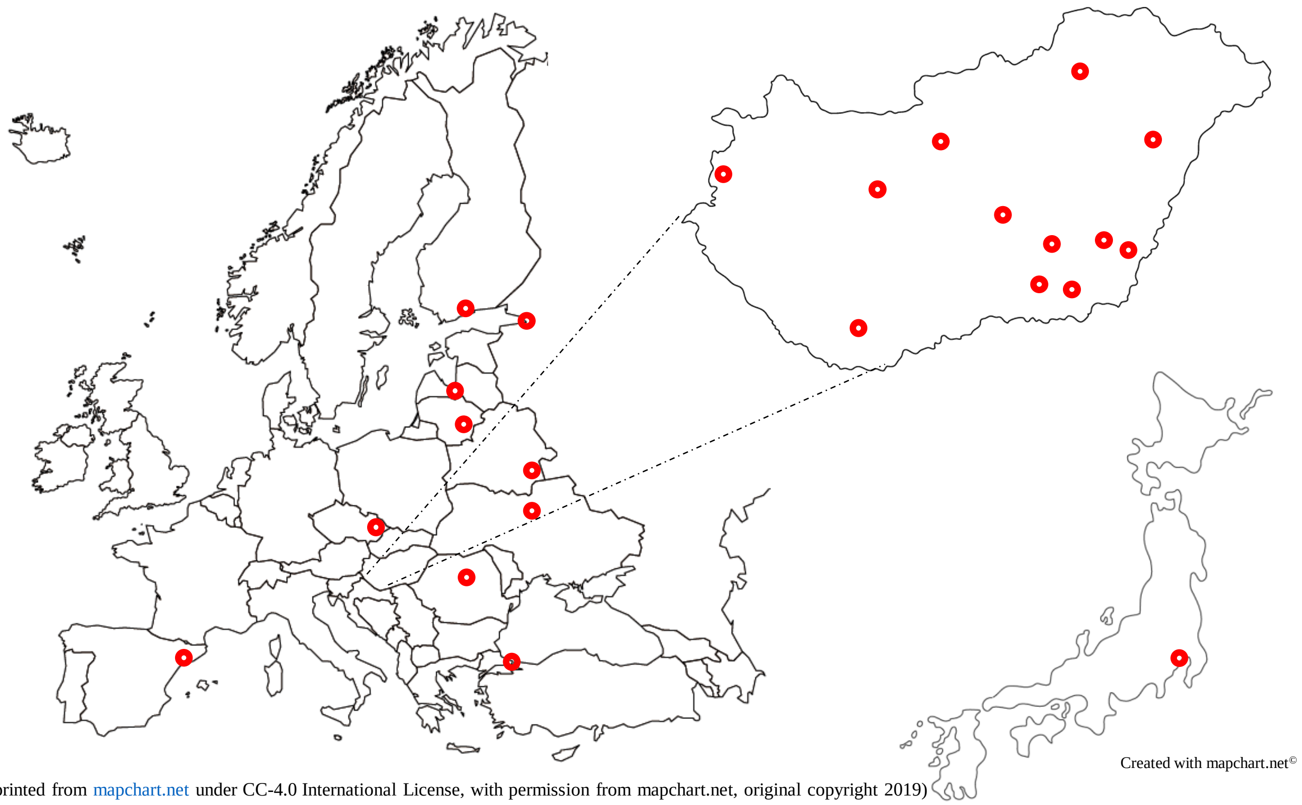

(Reprinted from mapchart.net under CC-4.0 International License, with permission from mapchart.net, original copyright 2019)

Country	City	Institute	No. of patients
Hungary	Pécs	First Department of Medicine, Medical School, University of Pécs	350
	Székesfehérvár	Szent György Teaching Hospital of County Fejér	190
	Szeged	First Department of Medicine, University of Szeged	224
		Second Department of Medicine, University of Szeged	27
	Budapest	Bajcsy-Zsilinszky Hospital	122
		Polyclinic of Hospitaller Brothers of Saint John of God	4
		Heim Pál National Institute of Pediatrics	1
		Department of Gastroenterology, Medical Center Hungarian Defence Forces	1
	Debrecen	Division of Gastroenterology, Department of Internal Medicine, University of Debrecen	72
		Institute of Surgery, University of Debrecen	7
	Békéscsaba	Dr. Réthy Pál Hospital of County Békés, Gastroenterology Department	40
	Gyula	Békés County Central Hospital-Pandy Kálmán Hospital, Dept. of Internal Med. and Gastroent.	22
	Szentes	Dr. Bugyi István Hospital	11
	Miskolc	Borsod-Abaúj-Zemplén County Hospital and University Teaching Hospital	12
	Kecskemét	Bács-Kiskun County Hospital	10
	Makó	Healthcare Center of County Csongrád	9
	Szombathely	Markusovszky University Teaching Hospital	9
Romania	Targu Mures	Mures County Emergency Hospital	41
Lithuania	Vilnius	Vilnius University Hospital Santariskiu Klinikos	31
Spain	Barcelona	Consorci Sanitari del Garraf, sant Pere de Ribes	30
Finland	Helsinki	Helsinki University Hospital, Department of Liver and Transplantation Surgery	27
Turkey	Istanbul	Hospital of Bezmialem Vakif University, School of Medicine	20
Russia	St. Petersburg	Saint Luke Clinical Hospital	18
Czech Republic	Ostrava	Centrum péče o zažívací trakt, Vítkovická nemocnice a.s.	11
Belarus	Gomel	Gomel Regional Clinical Hospital	8
Latvia	Riga	Pauls Stradins Clinical University Hospital, Gastroent., Hep. and Nutr. Centre	8
Ukraine	Kiev	Bogomolets National Medical University	8
Japan	Tokyo	Keio University	2
		**Total number of patients**	**1315**

Spp. Fig.5 Centre distribution of cases with chronic pancreatitis

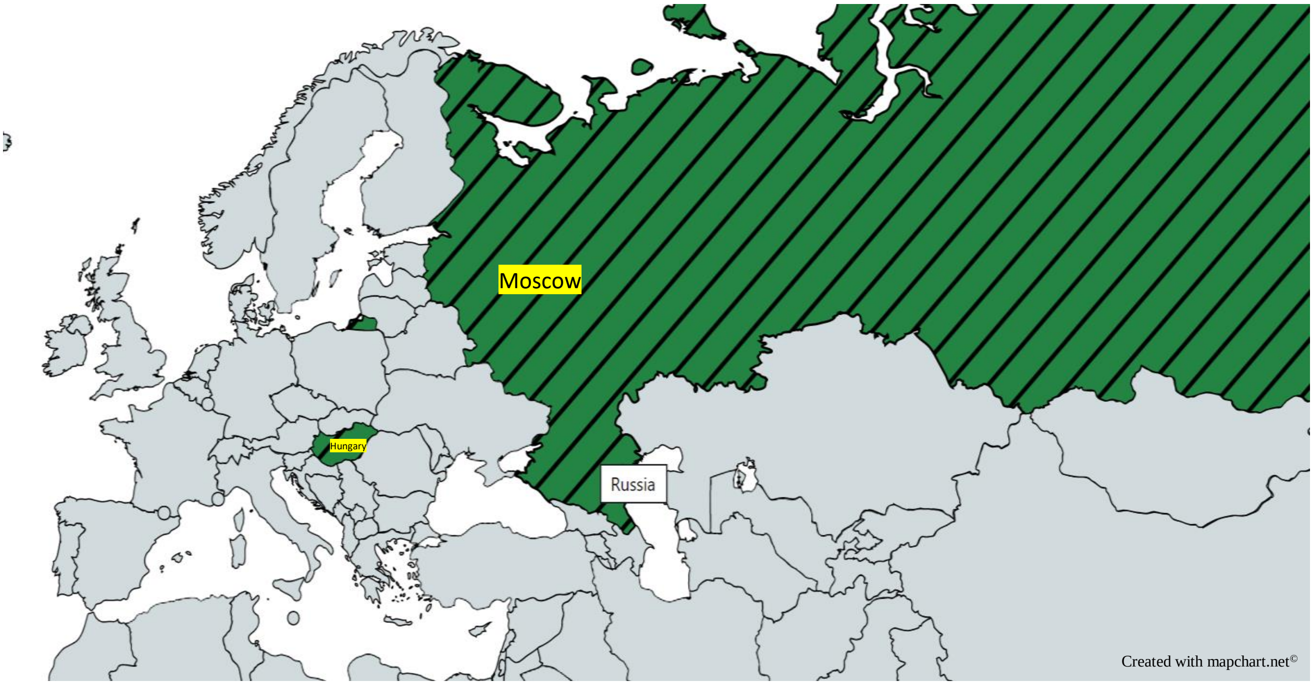

(Reprinted from mapchart.net under CC-4.0 International License, with permission from mapchart.net, original copyright 2019)

Country	City	Institute	No. of patients
Hungary	Pécs	First Department of Medicine, Medical School, University of Pécs, Pécs, Hungary	50
		Department of Surgery, Medical School, University of Pécs, Pécs, Hungary	22
	Székesfehérvár	Szent György University Teaching Hospital, Fejér County, Székesfehérvár, Hungary	33
	Szeged	First Department of Medicine, University of Szeged, Szeged, Hungary	77
		Department of Surgey, University of Szeged, Szeged, Hungary	29
		2nd Hospital, University of Szeged, Hungary	3
		Department of Emergency, University of Szeged, Szeged, Hungary	1
		Second Department of Medicine, University of Szeged, Szeged, Hungary	1
	Budapest	National Institute of Oncology, Budapest, Hungary	6
		Heim Pál National Institute of Pediatrics, Budapest, Hungary	4
		Department of Gastroenterology, Medical Center, Hungarian Defense Forces, Budapest, Hungary	2
		First Department of Surgery, Semmelweis University, Budapest, Hungary	2
		Bajcsy-Zsilinszky Hospital, Budapest, Hungary	1
		Hospital of the Hospitaller Order of Saint John of God, Budapest, Hungary	1
	Debrecen	Second Department of Medicine, University of Debrecen, Debrecen, Hungary	4
		Institute of Surgery, University of Debrecen, Debrecen, Hungary	8
	Békéscsaba	Dr. Réthy Pál Hospital, Békéscsaba, Hungary	4
	Gyula	Pándy Kálmán Hospital of Békés County, Gyula, Hungary	3
	Szentes	Dr. Bugyi István Hospital, Szentes, Hungary	3
	Kecskemét	Bács-Kiskun County Hospital, Kecskemét, Hungary	1
	Makó	Csongrád County Hospital, Makó, Hungary	1
	Miskolc	Borsod-Abaúj-Zemplén County Hospital, Miskolc, Hungary	4
	Nyiregyháza	Szabolcs-Szatmár-Bereg County Hospital, Nyíregyháza, Hungary	1
Russia	Moscow	A.S. Loginov Moscow Clinical Research and Practical Center, Moscow Healthcare Department, Moscow, Russia	93
		Semashko Central Clinical Hospital, Moscow, Russia	12
		Total number of patients	366

Detailed results of the novel experimental mouse model of reciditive acute pancreatitis

Representative hematoxylin-eosin-stained pancreas samples (Fig 6A) revealed histological differences between the AP, RAP, RAP2, RAP3 and RAP4 groups. Vacuolisation (Δ) was detected in the RAP, RAP2, RAP3 and RAP4 samples. Each scale bar represents 100µm.

Necrosis (Fig 6B) grades were elevated in all the CER-treated groups, with significant differences found between the AP and RAP (****$p<0.001$), AP and RAP2 (****$p<0.001$), AP and RAP3 (****$p<0.001$), or AP and RAP4 (****$p<0.001$) groups. n=3–5 animals/group. Data means ± SEM.

Edema (Fig 6B) in the CER-treated groups was elevated compared to the control groups (PS-treated), with significant differences found between the first AP induction and the RAP group (****$p<0.001$) and between the AP and RAP2 (****$p<0.001$), AP and RAP3 (****$p<0.001$), and AP and RAP4 (****$p<0.001$) groups as well. No more significant differences were found in the CER-treated groups. n=3–5 animals/group. Data means ± SEM.

Leukocyte infiltration (Fig 6B) grades were elevated in all the CER-treated groups, with significant differences found between the AP and RAP (****$p<0.001$), AP and RAP2 (****$p<0.001$), AP and RAP3 (****$p<0.001$), or AP and RAP4 (****$p<0.001$) groups. n=3–5 animals/group. Data means ± SEM.

Measurements revealed that serum amylase activity (Fig 6C) was most elevated in the case of AP. Serum amylase activity was significantly elevated in RAP compared to the controls (PS) or the RAP2, RAP3 or RAP4 groups (*$p<0.05$). Significant differences were found in serum amylase activity between the AP vs RAP2 or RAP3 (****$p<0.001$) and AP vs RAP4 (***$p<0.002$) groups. Significant differences were found between the RAP and RAP2 (**$p<0.01$) or RAP and RAP3 (#$p<0.03$) groups. The RAP4 group significantly differed from the AP group in amylase activity (****$p<0.001$), but not from the other groups. No significant differences were found between the AP and RAP, RAP3 and RAP, and RAP4 and RAP2 groups or between the RAP3 and RAP4 groups. n=4–5 animals/group. Data means ± SEM.

Serum IL-1β measurements (Fig 6D) revealed significantly elevated IL-1β levels in the RAP, RAP2, RAP3 and RAP 4 groups (AP and RAP4 (****$p<0.001$), AP and RAP (***$p<0.005$), AP and RAP2 (***$p<0.005$), and AP and RAP3 (*$p<0.05$)). CD3 (T cell, brown colour) staining showed significantly elevated CD3 levels in RAP3 and RAP4, AP and RAP3 (*$p<0.05$), and AP and RAP4 (***$p<0.005$) (Fig 6E). CD68 (macrophage, anti-macrosialin, brown colour) levels were significantly higher in the RAP 4 group (AP and RAP4 (*$p<0.05$)) (Fig 6F). Remarkable fibrosis was detected in the RAP2–RAP4 (blue staining ▲) samples. Each scale bar represents 100µm (Fig 6G).

https://imagej.nih.gov/ij/download.html

Supplementary Table 1. Data quality

Variable	Total (N^0 of pts)	Data available (N^0 of pts)	%
Sex	1435	1435	100,00%
Severity	1435	1435	100,00%
Mortality	1435	1435	100,00%
Length of hospitalization days	1435	1435	100,00%
Local pancreatic complications	1435	1428	99,51%
Fluid collection	1435	1428	99,51%
Pseudocyst	1435	1429	99,58%
Necrosis	1435	1429	99,58%
Diabetes mellitus as complication	1435	1429	99,58%
Other local complication	1435	1429	99,58%
Systemic complications organ failure	1435	1425	99,30%
Respiratory failure	1435	1425	99,30%
Heart failure	1435	1425	99,30%
Renal failure	1435	1425	99,30%
Other systemic complication	1435	1425	99,30%
Aetiology Biliary	1435	1435	100,00%
Aetiology Alcohol induced	1435	1435	100,00%
Aetiology Lipid metabolic disorder	1435	1434	99,93%
Aetiology Post-ERCP	1435	1241	86,48%
Aetiology Virus infection	1435	1435	100,00%
Aetiology Trauma	1435	1435	100,00%
Aetiology Drug induced	1435	1434	99,93%
Aetiology Congenital anatomical malformation	1435	1434	99,93%
Aetiology Cystic fibrosis	1435	1434	99,93%
Aetiology Gluten sensitive enteropathy	1435	1434	99,93%
Aetiology Genetic	1435	1435	100,00%
Aetiology Idiopathic	1435	1410	98,26%
Aetiology other	1435	1418	98,82%
Current smoking	1435	1427	99,44%
Current smoking amount	1435	1393	97,07%
Former smoking	1435	701	48,85%
Current alcohol consumption	1435	1429	99,58%
Former alcohol consumption	1435	665	46,34%
Diabetes mellitus	1435	1417	98,75%
Family history AP	1435	1435	100,00%
Family history CP	1435	1435	100,00%
Abdominal pain	1435	1432	99,79%
Abdominal pain type	1435	1148	80,00%
Abdominal pain location type	1435	1025	71,43%

Variable	Total (N^0 of pts)	Data available (N^0 of pts)	%
Abdominal pain location - right hypochondriac	1435	1432	99,79%
Abdominal pain location - left hypochondriac	1435	1432	99,79%
Abdominal pain location - epigastric region	1435	1432	99,79%
Abdominal pain location - right lumbar	1435	1432	99,79%
Abdominal pain location - left lumbar	1435	1432	99,79%
Abdominal pain location - umbilical region	1435	1432	99,79%
Abdominal pain location - right iliac	1435	1432	99,79%
Abdominal pain location - left iliac	1435	1432	99,79%
Abdominal pain location - hypogastric region	1435	1432	99,79%
Ad Local pancreatic complications	1435	1257	87,60%
Ad Fluid collection	1435	1256	87,53%
Ad Pseudocyst	1435	1256	87,53%
Ad Necrosis	1435	1257	87,60%
Ad Diabetes as complication	1435	1255	87,46%
Ad Other local complication	1435	1255	87,46%
Ad Systemic complications organ failure	1435	1423	99,16%
Ad Respiratory failure	1435	1423	99,16%
Ad Heart failure	1435	1423	99,16%
Ad Renal failure	1435	1423	99,16%
Ad Other systemic complication	1435	1423	99,16%
Ad Mortality	1435	1421	99,02%
Ad Intravenious fluid	1435	1339	93,31%
Abdominal pain length on admission hour	1435	1202	83,76%
Abdominal pain intensity	1435	727	50,66%
max CRP mg/L	1435	1402	97,70%
max WBC g/L	1435	1423	99,16%
max Lipase U/L	1435	1109	77,28%
max Amylase U/L	1435	1429	99,58%
ln LOH	1435	1435	100,00%
Ad CRP mg/L	1435	1176	81,95%
Ad WBC g/L	1435	1284	89,48%
Age at the time of admission	1435	1435	100,00%
BMI kg/m2	1435	1257	87,60%
age at first attack	1435	1284	89,48%
Ad Amylase U/L	1435	1311	91,36%
Ad Lipase U/L	1435	840	58,54%
LN Abdominal pain length on admission	1435	1202	83,76%
Ad Glasgow coma scale	1435	864	60,21%
Ad Red blood cell count T/L	1435	896	62,44%
Ad Hemoglobin g/L	1435	899	62,65%

Variable	Total (N⁰ of pts)	Data available (N⁰ of pts)	%
Ad Hematokrit %	1435	897	62,51%
Ad Thrombocyte g/L	1435	898	62,58%
Ad Glucose mmol/L	1435	1178	82,09%
Ad Blood urea nitrogen mmol/L	1435	1180	82,23%
Ad Creatinine umol/L	1435	1224	85,30%
Ad eGFR	1435	326	22,72%
Ad AST U/L	1435	746	51,99%
Ad LDH Lactate dehydrogenase U/L	1435	685	47,74%
Ad Calcium mmol/L	1435	589	41,05%
Ad Sodium mmol/L	1435	835	58,19%
Ad Potassium mmol/L	1435	837	58,33%
Ad Total protein g/L	1435	117	8,15%
Ad Albumin g/L	1435	117	8,15%
Ad Cholesterol mmol/L	1435	210	14,63%
Ad Triglyceride mmol/L	1435	274	19,09%
Ad ALT GPT U/L	1435	695	48,43%
Ad GGT U/L	1435	1072	74,70%
Ad Total bilirubin umol/L	1435	1148	80,00%
Ad Direct conjugated bilirubin umol/L	1435	409	28,50%
Ad ALP U/L	1435	1090	75,96%
Ad ESR mm/hour	1435	147	10,24%
Ad PCT Procalcitonin ng/ml	1435	138	9,62%
Ad Previous O2 therapy L/min.	1435	1071	74,63%
**Mean data quality**			**82.47%**

Ad: admission; **AP:** acute pancreatitis; **ALP:** alkaline phosphatase; **ALT:** alanine aminotransferase; **AST:** aspartate aminotransferase; **BMI:** body-mass index; **CP:** chronic pancreatitis; **CRP**: C-reactive protein; **ERCP:** endoscopic retrograde cholangiopancreatography; **GCS:** Glasgow Coma Scale; **GGT:** gamma-glutamyl transferase; **LDH:** lactate dehydrogenase; **RBC:** red blood cell count; **U:** unit; **WBC:** white blood cell;

Supplementary Table 2. Characteristics of patients with AP, ECP , and CP

	AP (non-ECP, non-CP)	ECP (non-CP)	CP
Sex (male%)	661/1199 (55.1%)	39/54 (72.2%)	45/62 (72.6%)
Moderately severe AP (%)	318/1199 (26.50%)	10/54 (18.5%)	16/62 (25.8%)
Severe (%)	57/1199 (4.80%)	0/54 (0%)	5/62 (8.1%)
Mortality (%)	24/1199 (2%)	0/54 (0%)	1/62 (1.6%)
Age (± SE in years)	1199/1199 (55.94±0.49)	54/54 (51.74±2.52)	62/62 (55.5±1.84)
LOH/days (SE)	1199/1199 (11.03±0.25)	54/54 (9.46±0.92)	62/62 (10.12±1.23)
Amylase (SE)	1110/1110 (1140±38.66)	53/53 (808±126)	51/51 (948±129)
Lipase (SE)	703/703 (2871±196.28)	40/40 (1807±314)	37/37 (2047±374)
CRP (SE)	1000/1000 (53.74±2.38)	49/49 (35±7.03)	51/51 (41±7.52)
